# Beyond the Obvious: Evaluating Incidence and Causes of False Positive Patent Foramen Ovale Diagnoses in Cryptogenic Ischemic Stroke—A Retrospective Analysis

**DOI:** 10.3390/jcdd12100400

**Published:** 2025-10-10

**Authors:** Raphael Phinicarides, Kira Berning, Houtan Heidari, Dominika Kanschik, Amin Polzin, Nikos Werner, Malte Kelm, Christian Jung, Kathrin Klein, Tobias Zeus, Shazia Afzal

**Affiliations:** 1 Division of Cardiology, Pneumology, and Vascular Medicine, University Hospital Düsseldorf, 40225 Dusseldorf, Germany; raphael.phinicarides@med.uni-duesseldorf.de (R.P.);; 2 Heartcenter Trier, Krankenhaus der Barmherzigen Brüder, 54292 Trier, Germanys.afzal@bbtgruppe.de (S.A.)

**Keywords:** PFO, cryptogenic ischemic stroke, echocardiography, false positive

## Abstract

(1) Background: Transesophageal echocardiography (TEE) is the gold standard for diagnosing patent foramen ovale (PFO) in cryptogenic ischemic stroke. However, false-positive diagnoses remain clinically relevant, exposing patients to unnecessary invasive procedures. (2) Methods: We retrospectively analyzed 346 patients with cryptogenic ischemic stroke who underwent TEE for PFO from 2012–2021. PFO was confirmed in 326 patients (94.2%), whereas 20 patients (5.8%, 95% CI 3.6–8.9%) were adjudicated as false positives during subsequent cardiac catheterization (intracardiac echocardiography, angiography, and inability to cross the interatrial septum). Univariable and multivariable logistic regression identified predictors of diagnostic accuracy. (3) Results: False-positive cases were associated with less frequent use of the mid-esophageal bicaval view (50% vs. 87%, *p* < 0.001) and absence of early bubble transit. Multivariable analysis confirmed the mid-esophageal bicaval view as an independent predictor of accurate diagnosis (OR 5.23, 95% CI 2.11–12.9, *p* < 0.001). (4) Conclusion: False-positive PFO diagnoses occur in ~6% of patients referred for closure. Three quality criteria—mid-esophageal aortic valve short axis, bicaval view, and bubble test with x-plane analysis—may improve diagnostic reliability. These hypothesis-generating findings require prospective validation and alignment with ASE/ESC guidelines to reduce unnecessary invasive procedures.

## 1. Introduction

Persistent foramen ovale (PFO) has garnered significant clinical attention in recent years, particularly among young adults who experience cryptogenic ischemic strokes, where the prevalence of this cardiac shunt can be as high as 50% [1,2]. Consequently, accurate identification of PFO is crucial for determining the underlying etiology, making imaging techniques essential in clinical practice. However, the comprehensive nature of current guidelines may hinder clinical implementation, leading to variable adherence and, in some cases, false-positive PFO diagnoses. Therefore, standardized TEE protocols with clear quality indicators are necessary for reliable PFO diagnosis [1,3].

A PFO is a remnant of embryonic circulation, and approximately 75% of the population experiences closure of the foramen ovale after birth as the lungs expand and left atrial pressure increases. If postnatal fusion of the septum primum and septum secundum does not occur, the PFO persists, creating a shunt between the left and right atria [3,4]. In many instances, a PFO remains undetected and is often diagnosed only when complications arise, such as paradoxical emboli [2,4,5].

Although transthoracic echocardiography (TTE) with a bubble test demonstrates reasonable sensitivity (46%) and high specificity (99%), transesophageal echocardiography (TEE) is regarded as the gold standard for effective PFO diagnosis [6,7,8].

However, emerging evidence has shown that in certain contexts, transcranial Doppler and contrast-enhanced transthoracic echocardiography (cTTE) may offer similar diagnostic accuracy to TEE, potentially serving as more accessible screening alternatives when TEE is not feasible [9].

During TEE, a flexible ultrasound probe is inserted through the esophagus to attain high-resolution images of the heart’s atria with reduced sound absorption [1]. The addition of a contrast medium or foamed NaCl solution during the bubble test is recommended to confirm the presence of a PFO [1,5].

International cardiac societies, such as the American Society of Echocardiography (ASE) and the European Society of Cardiology (ESC), provide detailed protocols for the systematic assessment of the interatrial septum in their guidelines [1,3].

Recently, these were further refined by the European Stroke Organisation (ESO), which introduced focused guidance for PFO evaluation in patients with cryptogenic stroke, placing particular emphasis on age stratification, neuroimaging correlation, and individual stroke risk profiles [10].

Recent European position papers provide an updated, multidisciplinary framework for the management of patients with PFO, covering general approach, risk stratification, and specific high-risk conditions such as decompression sickness, migraine, and arterial deoxygenation syndromes [11,12].

However, the comprehensive nature of these recommendations may not achieve the intended improvements in patient care. Therefore, we aimed to develop an algorithm for diagnosing PFO that is tailored for daily clinical practice while maintaining high-quality standards. This study identifies essential quality indicators during TEE examinations based on instances of false-positive PFO diagnoses, aiming to establish an optimal echocardiographic methodology for PFO screening.

Transesophageal echocardiography (TEE) remains the primary diagnostic tool for patent foramen ovale (PFO) assessment, as noted in the outdated 2012 guidelines [8]. However, there is comparatively limited existing data in the field of congenital heart defects, with diagnostic recommendations carrying an evidence level of C [13]. In contrast, the therapeutic landscape presents a different scenario. The correlation between cryptogenic stroke and elevated PFO prevalence is well established, leading to a recommendation grade of A and an evidence level of I for PFO closure [2,9].

Despite the central role of TEE in PFO workup, recent meta-analytic findings have underscored its limitations. Mojadidi et al. reported a sensitivity of 89.2% and specificity of 91.4% when validated against autopsy findings, illustrating that both missed and false-positive diagnoses can occur, particularly in the setting of suboptimal technique or anatomical variants [14].

Notably, a study conducted by Johansson et al. in 2010 specifically examined the occurrence of false-negative diagnoses associated with contrast injections in TEE among 14 patients [15]. Additionally, Rodrigues et al. emphasized the importance of performing an adequate Valsalva maneuver during the bubble test, pointing out that inadequate execution could lead to false-negative diagnoses [16].

While the focus of much of the existing literature has been on investigating false-negative diagnoses, particularly in the context of suboptimal Valsalva maneuvers or delayed bubble opacification, the issue of false-positive diagnoses presents an equally critical clinical challenge. A false-positive PFO diagnosis can lead to unnecessary and potentially high-risk percutaneous interventions, diverting resources and exposing patients to procedural complications without clinical benefit. Therefore, a deeper understanding of the factors contributing to these false-positive assessments is essential for enhancing patient safety and optimizing diagnostic algorithms. Our objective was to assess the incidence and causes of false-positive PFO diagnoses in patients with cryptogenic ischemic stroke.

## 2. Materials and Methods

**Figure 1:**
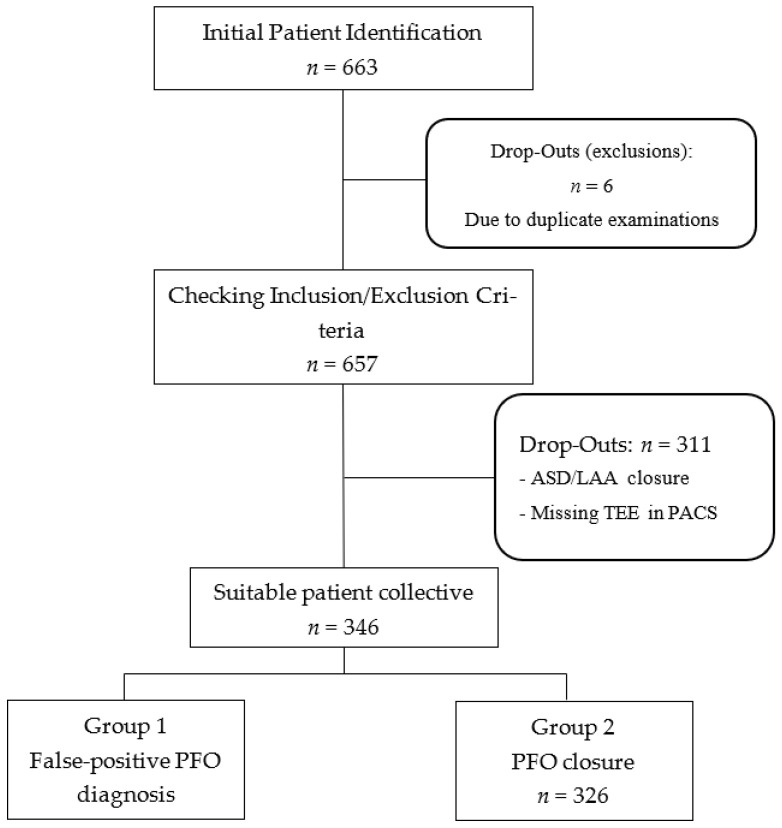
Flowchart of the screening process. Legend: ASD: Atrial septal defect, LAA: Left atrial appendage, TEE: transesophageal echocardiography, PACS: Picture Archiving and Communication System.

In this retrospective observational study, we included 346 patients who were diagnosed with PFO following a cryptogenic ischemic stroke from January 2012 to December 2021. The cohort was divided into two groups: those with confirmed PFO closure (n = 326) and those with a false-positive PFO diagnosis (n = 20). To ensure the highest standard of diagnostic accuracy, a definitive “false positive” diagnosis was operationally defined as the inability to confirm PFO presence during the subsequent cardiac catheterization procedure for attempted closure. The gold standard for confirmation during the interventional procedure was defined as a combination of intracardiac echocardiography, angiography, and the inability to cross the interatrial septum with a guidewire despite multiple attempts in different projections. In all 20 of these cases, the inability to cross the septum or a clear absence of a shunt via these direct visualization methods definitively ruled out the presence of a PFO. All invasive assessments were performed by the interventional cardiology team and were independent of the initial TEE report. Patient selection and study design are illustrated in Figure 1. The analysis of the stored TEE images was performed by two independent reviewers, both board-certified cardiologists with advanced TEE certifications from the European Society of Cardiology (ESC). These reviewers were not involved in the initial diagnostic TEE or the subsequent invasive procedure. To assess inter-observer agreement, a random subset of 50 TEE examinations was re-evaluated, showing a high kappa coefficient for the presence of a PFO (k = 0.85)

A total of 663 patients were initially identified, and after exclusions for duplicate examinations, ASD/LAA closure, and missing TEE data, the final cohort of 346 patients was divided into two groups: those with confirmed PFO closure and those with a false-positive PFO diagnosis.

Initial patient lists were generated and screened using Metek software HKWin version 1.148.2 (Metek Medizin Technik Komponenten GmbH, Elmshorn/Roetgen, Germany) and KardWin (Metek Medizin Technik Komponenten GmbH, Elmshorn/Roetgen, Germany). Further selection of patients who did not meet the inclusion and exclusion criteria was performed before a final subdivision into the two subgroups.

The analysis was divided into two parts. In the first part, we compared the stored image sequences from screening transesophageal echocardiograms (TEEs) with the diagnostic protocols outlined in current guidelines. In the second part, we evaluated the false-positive PFO diagnosis TEE images to identify indicators of error by correlating them with the image sequences of correctly diagnosed PFOs. This comparative approach aimed to investigate the relationship between examination sequences and false-positive PFO diagnosis, ultimately facilitating the identification of key quality indicators for effective diagnosis.

The transesophageal echocardiography (TEE) examinations analyzed in this study were conducted at both the Imaging Department of the Clinic of Cardiology, Pneumology, and Vascular Medicine, as well as the Clinic of Neurology. The procedures were performed by specially trained assistant physicians and cardiologists. We utilized various ultrasound machines, including the GE Logiq S8, Philips IE33, and Philips EPIQ 7, equipped with Philips X7-t2, X8-t2, and GE 6Tc Rs ultrasound probes.

Our analysis focused on imaging sequences of the interatrial septum (IAS). TEE examinations were triaged using the Picture Archiving and Communication System (PACS) for image data management. To enhance readability, we employed the commonly accepted English terminology for the probe positions, which included the upper esophageal short axis view (UE SAX), mid-esophageal short axis at the level of the aortic valve (ME AV SAX), mid-esophageal four-chamber view (ME 4CV), and mid-esophageal bicaval view (ME Bicaval). These probe positions, along with their respective angle settings, were used as key parameters for analysis, allowing for both 2D and 3D imaging as well as assessment of the bubble test.

The bubble test results were evaluated based on the timing of bubble crossing and the quantity of bubbles that crossed into the left atrium. Statistical analysis of the data was performed using IBM^®^ SPSS^®^ Statistics software, version 29.0 (IBM Corp., Armonk, NY, USA). Significance was assessed using the Pearson chi-square test or Fisher’s exact test, with a *p*-value of <0.05 deemed significant at a 95% confidence interval for all statistical tests conducted.

In addition to the univariable analysis, we conducted a multivariable logistic regression to identify independent predictors of a false-positive PFO diagnosis. The model included key variables that demonstrated statistical significance in the univariable analysis or were clinically relevant potential confounders, such as age, stroke severity, and the presence of anatomical variants like an atrial septal aneurysm (ASA) or a prominent Eustachian valve. The results of the multivariable analysis will be reported as odds ratios (OR) with 95% confidence intervals (CI) to provide a more nuanced understanding of the effect sizes.

The probe positions and angle recommendations used in this study are summarized in Table 1, in accordance with current guideline standards.

## 3. Results

The final cohort comprised 346 patients (mean age 52 ± 14 years). The patient population was divided into two groups: 20 patients (5.8%) with false-positive patent foramen ovale (PFO) diagnosis and 326 patients (94.2%) with confirmed PFO occlusions. There were no significant differences in cardiovascular risk factors, including arterial hypertension, diabetes mellitus, hyperlipidemia, nicotine use, and positive family history, between the two groups. Baseline demographic and clinical characteristics, probe positions, and bubble test results by subgroup are detailed in Table 2.

A total of 346 patients with a mean age of 52 ± 14 years were included in the study. The patient population was divided into two groups: 20 patients (5.8%) with false-positive patent foramen ovale (PFO) diagnosis and 326 patients (94.2%) with confirmed PFO occlusions. There were no significant differences in cardiovascular risk factors, including arterial hypertension, diabetes mellitus, hyperlipidemia, nicotine use, and positive family history, between the two groups.

Among the study population, 243 patients (71.1%) had cerebral ischemia, 84 (24.6%) had transient ischemic attacks (TIA), and 15 (4.4%) presented with paradoxical embolism. The relative incidence of these conditions was similar between both groups, except for paradoxical embolism; all 15 cases were assigned to the PFO group.

**Figure 2:**
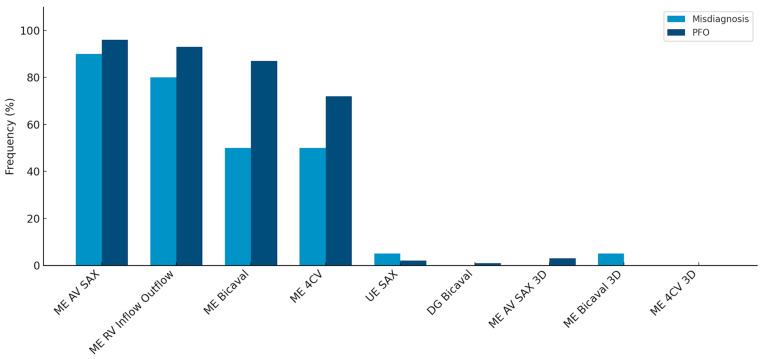
Probe position frequencies in PFO diagnostics.

When evaluating adherence to guidelines across the entire cohort, it was noted that imaging planes in the mid-esophagus were used significantly more frequently than modified probe positions in the upper and transgastric planes (71–96% vs. 1–2%). The mid-esophageal aortic valve short axis view (ME AV SAX) was employed in 95% of TEE diagnoses, while the mid-esophageal right ventricular inflow and outflow view (ME RV Inflow Outflow) was observed in 92% of examinations. Three-dimensional imaging of the interatrial septum (IAS) was infrequently performed (0–3%). To align with guidelines, the bubble test was utilized in 98.8% of the 346 examinations, with only four cases omitting this test (Table 2). Overall use of probe positions is visualized in Figure 2.

Comparison of the most frequently used probe positions and 3D recordings in the false-positive and PFO subgroups. Frequencies are listed in descending order from left to right, starting with standard views and proceeding to less frequently used three-dimensional settings. This figure highlights the statistically significant difference in the use of the mid-esophageal bicaval (ME Bicaval) view and four-chamber view (ME 4CV) between the two groups, while also showing the low overall utilization of 3D imaging in both cohorts.

Comparing the two subgroups revealed a lower overall frequency of diverse probe positions in the false-positive PFO diagnosis group. Specifically, in this group, the IAS was predominantly presented in the mid-esophageal short axis view (ME AV SAX), observed in 90% of cases.

Detailed frequencies of probe views and bubble test performance across both groups are provided in Table 2.

This setting was also prevalent in the PFO group (96%). However, a statistically significant difference of 21% in the frequency of the four-chamber view was noted between the groups (50% vs. 71%, *p* = 0.04). A highly significant difference (*p* < 0.001) was observed in the bicaval view in the mid-esophagus, albeit with a small effect size (V = 0.2).

Multivariable logistic regression revealed that the use of the mid-esophageal bicaval view was an independent predictor of an accurate PFO diagnosis (OR 5.23, 95% CI 2.11–12.9; *p* < 0.001). This confirms the findings from our univariable analysis and highlights the clinical importance of this specific view in the diagnostic protocol. Conversely, the mid-esophageal short axis view, while frequently used, did not show a significant association with diagnostic accuracy in the adjusted model (OR 2.99, 95% CI 0.98–9.11; *p* = 0.21).

The bubble test was conducted in 95% of the false-positive PFO diagnosis group and in 99.1% of the control group (*p* = 0.12). In the false-positive PFO diagnosis group, visualization during the bubble test occurred in 80% using the short axis view and in 30% using the bicaval view. In comparison, the control group demonstrated frequencies of 63% and 47%, respectively. IAS imaging in x-plane mode was similarly low in both groups (35% vs. 33%). In 88% of all bubble tests, the first microbubbles were detected in the left atrium (LA) within the first six cardiac cycles; this criterion was met in only two cases (10%) within the false-positive PFO diagnosis group. Additionally, two-thirds (65%) of bubble tests in the false-positive PFO diagnosis group showed no bubbles in the LA during recorded image sequences, contrasted with only 2% in the control group, which was highly significant (*p* < 0.001). In this subgroup, more than six microbubbles appeared in the LA in 79.5% of tests, demonstrating highly significant differences (*p* = 0.02).

## 4. Discussion

Our study demonstrates that approximately 6% of patients with cryptogenic stroke initially diagnosed with PFO on TEE were subsequently found to have no defect during invasive evaluation. This non-negligible false-positive rate emphasizes the importance of standardized imaging protocols to avoid unnecessary catheterization.

The strongest predictor of accurate diagnosis was the consistent use of the mid-esophageal bicaval view, which directly visualizes the anterosuperior portion of the fossa ovalis. Reliance on a single frequently used view, such as the aortic valve short axis, was insufficient and contributed to false-positive PFO diagnosis. These findings support the implementation of structured checklists incorporating key probe positions and bubble test execution, thereby reducing operator dependence.

Aligning our proposed quality criteria with existing ASE and ESC recommendations could enhance their clinical applicability. For example, guideline-based standards already emphasize multiple views, adequate Valsalva maneuvers, and contrast-enhanced testing; our analysis highlights which elements are most discriminative in preventing false positives. Beyond TEE, confirmatory non-invasive modalities such as cTTE or TCD should be integrated when TEE findings are ambiguous, providing an additional safeguard before referral to invasive closure.

**Figure 3:**
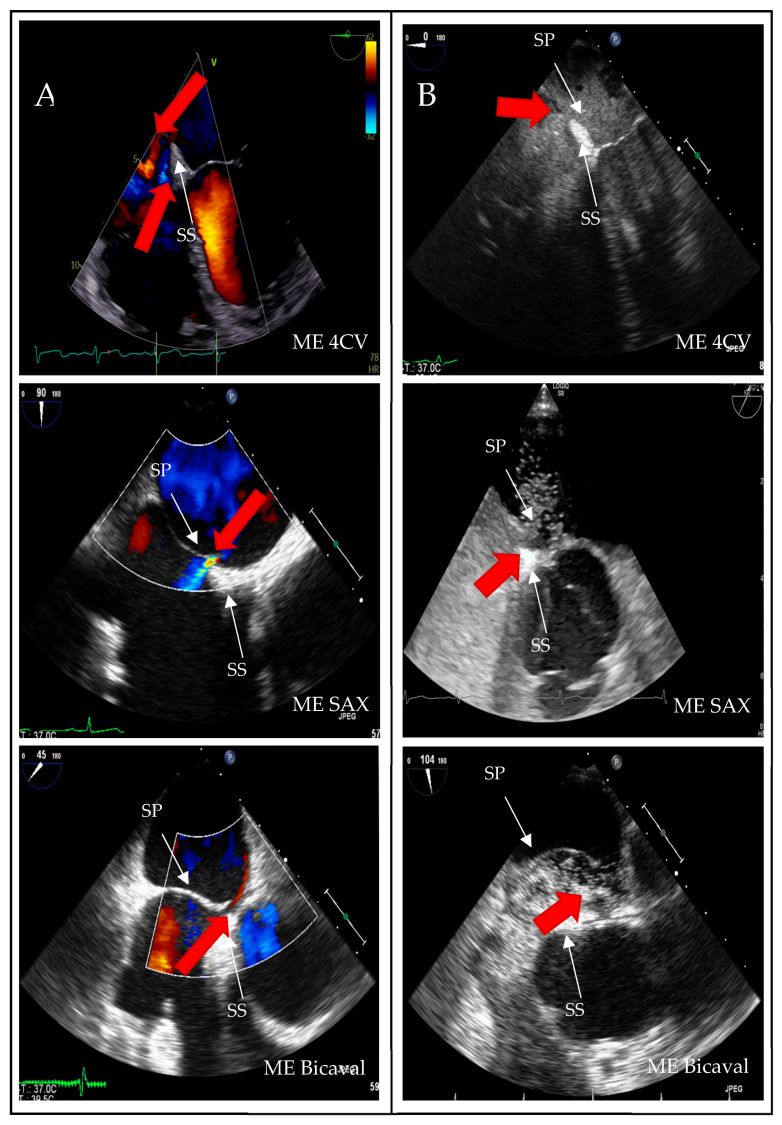
Two-dimensional TOE recordings of an IAS with PFO. Shown is a short circuit between the RA and LA in ME 4CV, ME SAX, ME Bicaval image planes (**A**) in the color Doppler and (**B**) in the bubbles test; the red arrow indicates the direction of the short circuit. ME: midesophageal, SAX: short axis, 4CV: four chamber view, SP: septum primum, SS: septum secundum.

A structured approach could transform PFO diagnosis from a highly operator-dependent procedure into a reproducible, high-quality standard, particularly beneficial for less experienced operators. Given the results presented, we investigated whether precise quality indicators could enhance the reliability of PFO diagnosis in the context of transesophageal echocardiography (TEE). Standard TEE views demonstrating PFO shunting are shown in Figure 3.

Furthermore, the integration of alternative imaging modalities could serve as valuable confirmatory steps before proceeding to invasive PFO closure. Contrast-enhanced transthoracic echocardiography (cTTE) and transcranial Doppler (TCD) with bubble contrast are non-invasive methods that can effectively screen for right-to-left shunts. While TEE remains the gold standard for detailed anatomical assessment, a discordant result between TEE and a non-invasive modality, or a high clinical suspicion of PFO despite an ambiguous TEE, could trigger a re-evaluation or stricter adherence to advanced TEE protocols. This hierarchical approach, utilizing non-invasive screening as a complementary tool, could further reduce the incidence of false-positive diagnoses and prevent unnecessary invasive procedures, thereby enhancing patient safety and resource allocation.

Among the initial patient population of 663 referred for PFO diagnosis from smaller hospitals or office-based physicians, only approximately half (n = 346) underwent pre-interventional screening TEE at the UKD facilities. Over a data collection period of 10 years, 23 cases of false-positive PFO findings were identified during elective interventional procedures in the cardiac catheterization laboratory. Of these, the echocardiographic examinations of 20 patients were included in the present analysis, representing the false-positive group in comparison with the 326 patients in the confirmed PFO group.

Sub-study 1: In the initial analysis of the TEE examinations, the complete dataset was examined. Common probe positions ranged from 70% to 95% prevalence at the mid-esophageal level. However, visualization of the interatrial septum (IAS) in the four-chamber view was often inadequate, as the IAS was not fully visible. Although three-dimensional (3D) imaging is now standardized for valve assessment and is recommended for PFO diagnosis in ambiguous cases, 3D imaging of the IAS was performed in only 4% of the 346 TEEs analyzed. Contrast echocardiography utilizing bubble testing was conducted in 99% of the cases within the dataset.

Sub-study 2: This sub-study aimed to compare the echocardiographic images of patients with false-positive PFO diagnoses against those with accurate diagnoses to determine the necessary steps and views in TEE for confirming a PFO diagnosis. Overall, there was less variability in the probe settings used to visualize the interatrial septum (IAS) within the false-positive group compared to the accurate diagnosis group. In the false-positive PFO diagnosis group, IAS imaging predominantly occurred in the mid-esophageal short axis (80–90%).

These findings align with previously published observations on procedural pitfalls, such as misaligned imaging planes, suboptimal probe angulation, or lack of experience with shunt visualization during TEE [17].

The PFO is situated in the anterior–superior part of the fossa ovalis [3]. Visualization of the anterior fossa ovalis limb on TEE is typically achieved only in the short axis at the level of the aortic valve (mid-esophageal aortic valve short axis view, ME AV SAX). However, statistical analysis showed no significant correlation between this probe position and diagnostic accuracy (*p* = 0.21; odds ratio [OR] 2.99). In contrast, the mid-esophageal bicaval view (ME Bicaval) demonstrated a strong correlation with accurate diagnoses (*p* < 0.001; OR 5.23), as it presents the superior aspect of the fossa ovalis where the PFO is located [3]. Additionally, the four-chamber view was found to be statistically significant (*p* = 0.04; OR 1.12); however, its relevance in PFO diagnostics is questionable as it primarily images the posterior region of the fossa ovalis limbus, which is less important for PFO identification.

The finding from our multivariable analysis that the mid-esophageal bicaval view is an independent predictor of accurate diagnosis is particularly significant. It underscores the critical role of specific imaging planes in revealing the anterior–superior location of the PFO, an area often not fully visualized in standard four-chamber or short-axis views. This highlights the importance of a structured, comprehensive approach to TEE rather than relying on a single, frequently-used view. These results suggest that while operator experience is crucial, its impact can be enhanced by strict adherence to a standardized checklist of views, particularly in the setting of ambiguous findings, as this reduces the risk of diagnostic errors stemming from incomplete anatomical assessment.

These recommendations are consistent with the latest expert consensus on the treatment of PFO, which emphasizes individualized patient selection, optimal procedural techniques, and structured follow-up to maximize clinical benefit [17].

Previous studies indicate that methods such as the bubble test can achieve sensitivity levels of 96% when optimally performed [15]. Given the anterior–superior location of the PFO within the fossa ovalis, we also sought to explore whether probe position influenced the outcomes of the bubble test. No correlations were found between probe position and diagnostic group, whether in conventional imaging (*p* = 0.13–0.71) or in x-plane mode (*p* = 0.71–1.0). In conventional 2D imaging, both the mid-esophageal short axis (*p* = 0.13) and bicaval views (*p* = 0.13) exhibited better correlation evidence. However, x-plane representation during the bubble test did not significantly affect diagnostic confidence in this analysis, which may be attributed to the low frequency of observations in the PFO group.

As outlined in the guidelines, the appearance of microbubbles in the left atrium (LA) within the first six cardiac cycles is highly significant (*p* = <0.001). In 90% of the false-positive PFO diagnosis group, this dynamic was absent, while early crossing was observed in over 90% of the PFO cases. Moreover, statistical analyses support a correlation between the number of bubbles crossed and PFO diagnosis, indicating that the absence of bubble crossing in the LA strongly suggests a negative PFO diagnosis (*p* = <0.001). Unexpectedly, no positive bubble tests were detected in seven TEE examinations despite the presence of PFO. Retrospective image analysis does not conclusively determine whether there was no bubble crossing or if that aspect of the examination was not recorded.

Correctly diagnosed PFOs reliably showed inflow of more than six microbubbles within the first six cardiac cycles in 98.8% of cases, compared to only 15% in the false-positive group.

However, false negative results during the bubble test can also occur, particularly in cases of insufficient Valsalva or delayed right atrial opacification. In this context, clinicians should be aware of limitations in sensitivity even with proper technique [18].

False positive PFO diagnoses could be attributed to intrapulmonary shunts during late inflow. TEE studies from the false diagnosis group indicated a clustered occurrence of spontaneous echo contrast in the left atrium, which may simulate a positive bubble test. In 2D imaging, this increased echogenicity manifests as a “fog” in the blood, attributed to heightened erythrocyte aggregation [16].

In eleven of the 20 false-positive PFO diagnosis cases, spontaneous contrast was observed in the left atrium (LA). The timing of microbubble appearance in LA serves as a general guideline but is not a reliable indicator. According to Soliman et al., there is significant temporal overlap in the occurrence of bubbles in LA from both a PFO and a proximal interpulmonary shunt [19]. As illustrated, the presence of interatrial septum (IAS) hypermobility significantly influences the timing of bubble crossover. Delayed crossing may result from residual fetal structures in the right atrium, such as the Eustachian valve and the Chiari network. In 5% of patients with PFO, bubbles were detected in LA after six heartbeats.

**Figure 4:**
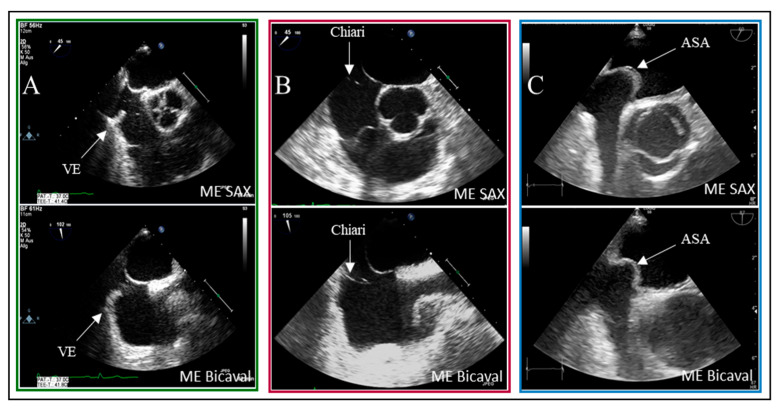
Two-dimensional TOE recordings of fetal anatomical structures as risk factors for PFO. Shown in the ME SAX and ME Bicaval views are (**A**) a Eustachian valve at the ostium of the inferior vena cava, (**B**) a Chiari network as a ribbon, fenestrated membrane in the right atrium, and (**C**) an atrial septal aneurysm with a protrusion into the right and left atrium. VE: Valvula Eustachii, ASA: Atriales Septumaneurysma, ME: Midesophageal, SAX: short axis.

Examples of residual fetal structures are shown in Figure 4.

This analysis revealed a statistical association between high-grade shunt intensity and PFO diagnosis in the context of IAS hypermobility (*p* = 0.02). In contrast, remnants of fetal circulation did not significantly impact shunt flow intensity. However, delayed contrast filling of the right atrium remains visible in the imaging sequences, which is critical for the bubble test, as indicated by Johansson et al. [15].

Accurate execution of the bubble test is essential and may require repetition in instances where findings are inconsistent. Interventional PFO exclusions benefit from having injections into the femoral vein, which provides the highest sensitivity, particularly when enhanced by the Eustachian valve.

Historically, the understanding of PFO has evolved significantly. Initially regarded as a benign anatomical variant, it is now recognized as a potential conduit for paradoxical embolism and a target for intervention in cryptogenic strokes. This shift underscores the growing importance of robust diagnostic algorithms and therapeutic clarity [19].

These major criteria in TEE are necessary to confirm the diagnosis based on the results and the anatomical location of the PFO, to avoid invasive PFO exclusion in the future.

**Figure 5:**
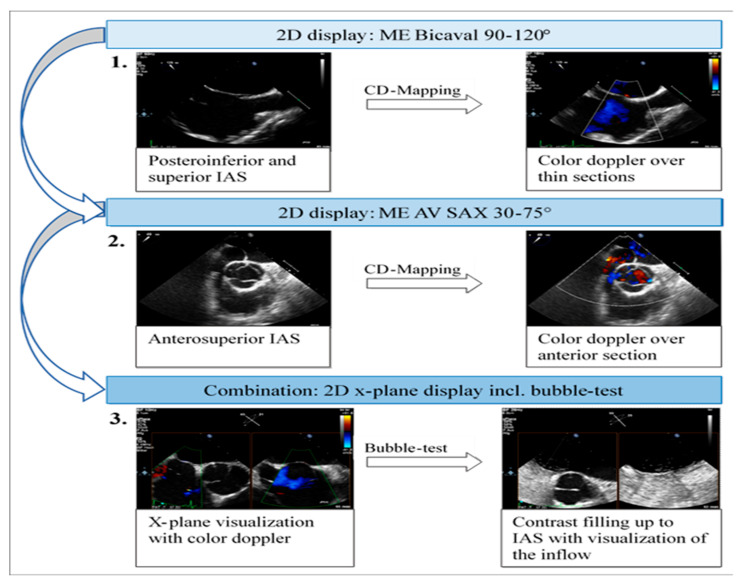
Illustration of an expanded diagnostic checklist incorporating the three key quality criteria (ME AV SAX, ME Bicaval, bubble test with x-plane) together with two adjunctive elements (upper esophageal view, 3D IAS imaging). 2D: two-dimensional, ME: midesophageal, AV: aortic valve, SAX: short axis, IAS: interatrial septum, 3D: three-dimensional.

In practical terms, systematic application of these quality criteria could prevent a substantial number of unnecessary invasive referrals. In our cohort, 20 of 346 patients (≈6%) underwent cardiac catheterization only to have PFO excluded. A structured checklist (see Figure 5) incorporating bicaval and aortic valve short axis views together with bubble testing in x-plane could have avoided most of these cases, sparing patients the procedural risk and conserving healthcare resources.

### Limitations

Several limitations of this study warrant acknowledgment. First, its retrospective, single-center design inherently introduces potential for selection bias and limits the generalizability of our findings to other populations and clinical settings. Second, while we implemented rigorous methods for TEE re-evaluation, the original TEE examinations were not standardized across all operators over the entire study period, which could introduce variability in image acquisition. Furthermore, the reviewers re-examining the archived TEE sequences were not blinded to the final invasive outcome (true vs. false-positive PFO). Despite high inter-observer agreement, this introduces potential review bias. We acknowledge that in a retrospective analysis, it is challenging to ascertain whether a specific part of the examination was truly not performed or simply not recorded in the archived sequences. Finally, this study focuses solely on diagnostic accuracy and does not include long-term outcome data for patients with confirmed PFO closure versus those with false-positive diagnoses. Future prospective, multi-center studies utilizing standardized TEE protocols and blinded image analysis would be necessary to validate our proposed quality criteria and assess their impact on long-term clinical outcomes.

## 5. Conclusions

To develop a strategy for reducing false positive diagnoses of patent foramen ovale (PFO), we retrospectively analyzed 346 TEE examinations at the University Hospital Düsseldorf. Although color Doppler mapping and the bubble test were performed in all examinations, not all recommendations outlined in the guidelines were adequately followed.

Visualization of the interatrial septum (IAS) from the upper esophagus or gastric views is rarely achieved in PFO diagnostics, and three-dimensional imaging of the IAS is infrequent in clinical practice. The sequential acquisition of IAS images primarily occurred from the mid-esophagus in the two-dimensional short-axis and bicaval views. For efficient and reliable IAS visualization, the bubble test should be conducted in the mid-esophagus utilizing x-plane mode with the short axis (30–75°) and the bicaval view (90–120°). This approach facilitates parallel visualization of the anterosuperior portion of the fossa ovalis and the inflow of bubbles via the superior vena cava (SVC).

In conclusion, our study identifies a non-negligible incidence of false-positive PFO diagnoses in patients with cryptogenic ischemic stroke, primarily driven by suboptimal TEE imaging protocols. We propose three key quality criteria as a hypothesis for improving diagnostic accuracy: the routine use of 2D imaging in the mid-esophageal aortic valve short axis, the mid-esophageal bicaval view, and the meticulous execution of the bubble test with simultaneous x-plane mode analysis. These findings underscore the need for standardized TEE guidelines and comprehensive operator training. While these criteria show significant promise in mitigating diagnostic pitfalls, they are hypothesis-generating and require prospective validation in larger, multi-center studies to confirm their efficacy and impact on patient outcomes.

## Figures and Tables

**Table 1:** TEE workflow evaluation for PFO assessment.

	Probe Position	Angle	3D-Angle	Illustrated IAS
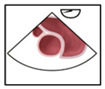	UE Short Axis View	0° 15° 30° 45°		superiores IAS with SS
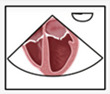	ME 4-Chamber View	0° 15° 30°	0–20°	posteriores IAS
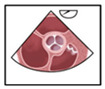	ME AV SAX View	30° 45° 60° 75°	30–60°	anterosuperiores IAS, Overlap of SP and SS
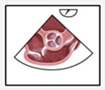	ME RV Inflow-Outflow View	55° 70°		Superiores IAS
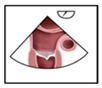	ME Modified Bicaval TV View	55° 70°		mid IAS
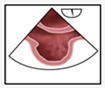	ME Bicaval View	90° 105° 120°	90–120°	posteroinferiores and superiores IAS
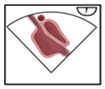	DG Bicaval View	100° 120°	100–120°	inferosuperiores IAS

IAS: interatrial septum, 3D: three dimensional, UE: upper esophageal, ME: midesophageal, AV: aortic valve, SAX: short axis, RV: right ventricular, TV: tricuspid valve, DG: deep transgastric, SP: septum primum, SS: septum secundum [1].

**Table 2:** Patient characteristics, probe positions, Comparison of the subgroups regarding the probe positions performed and bubble test results by subgroup.

Characteristics	Total n = 346	Misdiagnosis n = 20	PFO n = 326	*p*-Value
Age, Years	52 ± 14	54 ± 15	52 ± 14	0.54
Gender: Male Female	201 (58.8) 145 (41.2)	14 (72.2) 6 (27.8)	188 (58.0) 136 (42)	0.23 0.23
Hight, cm Weight, kg BMI, kg/m^2^	176 ± 10 83 ± 18 26.7 ± 4.9	176 ± 11 84 ± 18 27.1 ± 4.9	176 ± 10 83 ± 19 26.7 ± 4.9	0.76 0.71 0.74
**Cardiovascular RF**				
Artrial Hypertension	149 (56.4)	8 (44.4)	141 (43.5)	0.94
Diabetes mellitus: Typ I Typ II	4 (1.2) 26 (7.6)	0 (0) 2 (11.1)	4 (1.2) 24 (7.4)	0.67 0.64
Hyperlipidemia	108 (31.6)	6 (33.3)	102 (31.5)	0.87
Nicotinabus: Ex smoker Smoker	44 (12.9) 59 (17.3)	5 (27.8) 4 (22.2)	39 (12) 55 (17)	0.66 0.53
Family history	20 (5.8)	2 (11.1)	18 (5.6)	0.33
**Ischemic Insulte**				
Stroke TIA Other location Number Strokes	243 (71.1) 88 (24.6) 15 (4.4) 1 ± 1	14 (77.8) 6 (22.2) 0 (0) 2 ± 1	229 (70.7) 80 (24.7) 15 (4.6) 1 ± 1	0.52 1.00 1.00 0.03 *
**Probe position**				
**2D**				
UE SAX ME 4CV ME AV SAX ME RV Inflow-Outflow ME Bicaval DG Bicaval		1 (5) 10 (50) 18 (90) 16 (80) 10 (50) 0 (0)	5 (1.5) 234 (71) 313 (96) 303 (92.9) 283 (86.8) 4 (1.2)	0.30 0.04 * 0.21 0.06 <0.001 *** 1.00
**3D**				
ME 4CV 3D ME AV SAX 3D ME Bicaval 3D		0 (0) 0 (0) 1 (5)	1 (0.3) 9 (2.8) 1 (0.3)	1.00 1.00 0.11
**Bubble-Test**				
Bubble-Test ME 4CV ME SAX ME Bicaval		19 (95) 2 (10) 16 (80) 6 (30)	323 (99.1) 30 (9.2) 206 (63.2) 154 (47.2)	0.21 0.71 0.13 0.13
**Bubble-Test**				
**2D**				
ME 4CV ME SAX ME Bicaval	32 (9.2) 222 (64.1) 160 (46.2)	2 (10) 16 (80) 6 (30)	30 (9.2) 206 (63.2) 154 (47.2)	0.71 0.13 0.13
**2D-x-Plane**				
ME 4CV ME SAX ME Bicaval	13 (3.8) 42 (12.1) 60 (17.3)	0 (0) 3 (15) 4 (20)	13 (4) 39 (12) 56 (17.2)	1.00 0.72 0.76
**Detection**				
Heart cycles: n.a. <6 cycles >6 cycles	20 (5.8) 306 (88.4) 20 (5.8)	13 (65) 2 (10) 5 (25)	7 (2.1) 304 (93.3) 15 (4.6)	<0.000 *** <0.000 *** <0.001 ***
Bubble degree: none mild moderate severe	20 (5.8) 64 144 118	13 (65) 4 (20) 3 (15) 0 (0)	7 (2.1) 60 (18.4) 141 (43.3) 118 (36.2)	<0.000 *** 0.67 0.025 ** 0.002 **
Bubble-Shunt FD-Shunt	181 154	n.a. n.a.	181 (55.5) 154 (47.2)	

PFO: Patent Foramen Ovale. 2D: two dimensional. ME: midesophageal. AV: aortic valve. SAX: short axis. RV: right ventricular. 4CV: four chamber view. UE: upper esophageal. DG: deep transgastric. 3D: tree dimensional. FD: color doppler; * *p*-value < 0.05. ** *p*-value < 0.03. *** *p*-value < 0.001. Degree classification: mild = <6 Bubbles. moderat = 6–25 Bubbles. severe = >25 Bubbles.

## Data Availability

The data presented in this study are available on reasonable request from the corresponding author. The data are not publicly available due to privacy and legal restrictions under the EU General Data Protection Regulation (GDPR) and institutional policies of the University Hospital Düsseldorf.

## Abbreviations

The following abbreviations are used in this manuscript:

2D	Two-Dimensional
3D	Three-Dimensional
ASA	Atrial septal aneurysm
ASD	Linear dichroism
AV	Aortic valve
cTTE	Contrast enhanced transthoracic echocardiography
DG	Deep transgastric
ESO	European Stroke Organisation
ESC	European Society of Cardiology
FD	Color Doppler (Farbdoppler)
IAS	Interatrial septum
ICD	International Classification of Diseases
LA	Left atrium
LAA	Left atrial appendage
LV	Left ventricle
ME	Mid-esophageal
OPS	Operation and procedure coding system (Germany)
OR	Odds ratio
PACS	Picture archiving and communication system
PFO	Patent foramen ovale
RA	Right atrium
RV	Right ventricle
SAX	Short axis view
SP	Septum primum
SS	Septum secundum
SVC	Superior vena cava
TEE	Transesophageal echocardiography
TIA	Transient ischemic attack
TTE	Transthoracic echocardiography
UE	Upper esophageal
VE	Valvula Eustachii
